# Evaluation of the Effectiveness of the Biopreparation in Combination with the Polymer γ-PGA for the Biodegradation of Petroleum Contaminants in Soil

**DOI:** 10.3390/ma15020400

**Published:** 2022-01-06

**Authors:** Katarzyna Wojtowicz, Teresa Steliga, Piotr Kapusta, Joanna Brzeszcz, Tomasz Skalski

**Affiliations:** 1 Oil and Gas Institute—National Research Institute, Lubicz 25 A, 31-503 Krakow, Poland; teresa.steliga@inig.pl (T.S.); piotr.kapusta@inig.pl (P.K.); joanna.brzeszcz@inig.pl (J.B.); 2 Biotechnology Centre, Silesian University of Technology, Krzywoustego 8, 44-100 Gliwice, Poland; tomasz.skalski@polsl.pl

**Keywords:** biodegradation, petroleum contaminants, biopreparation, inoculation, polyglutamic acid, ecotoxicology

## Abstract

Biodegradation is a method of effectively removing petroleum hydrocarbons from the natural environment. This research focuses on the biodegradation of aliphatic hydrocarbons, monoaromatic hydrocarbons such as benzene, toluene, ethylbenzene, and all three xylene isomers (BTEX) and polycyclic aromatic hydrocarbons (PAHs) as a result of soil inoculation with a biopreparation A1 based on autochthonous microorganisms and a biopreparation A1 with the addition of γ-PGA. The research used biopreparation A1 made of the following strains: *Dietzia* sp. *IN133*, *Gordonia* sp. *IN138* *Mycolicibacterium frederiksbergense* *IN53*, *Rhodococcus erythropolis* *IN119*, *Rhodococcus* sp. *IN136* and *Pseudomonas* sp. *IN132*. The experiments were carried out in laboratory conditions (microbiological tests, respirometric tests, and in semi-technical conditions (*ex-situ* prism method). The biodegradation efficiency was assessed on the basis of respirometric tests, chromatographic analyses and toxicological tests. As a result of inoculation of AB soil with the biopreparation A1 within 6 months, a reduction of total petroleum hydrocarbons (TPH) (66.03%), BTEX (80.08%) and PAHs (38.86%) was achieved and its toxicity was reduced. Inoculation of AB soil with the biopreparation A1 with the addition of γ-PGA reduced the concentration of TPH, BTEX and PAHs by 79.21%, 90.19%, and 51.18%, respectively, and reduced its toxicity. The conducted research has shown that the addition of γ-PGA affects the efficiency of the biodegradation process of petroleum pollutants, increasing the degree of TPH biodegradation by 13.18%, BTEX by 10.11% and PAHs by 12.32% compared to pure biopreparation A1.

## 1. Introduction

Oil mining exploits valuable, non-renewable resources of natural gas and crude oil, and also contributes to the degradation of the biologically active surface of the earth. The works carried out in the oil and gas mining are accompanied by the necessity to collect and manage large amounts of waste materials, which were stored in waste pits in the years 1920–1950 [1]. In terms of chemicals, these wastes are diverse, multi-component systems that changed during deposition and initial treatment processes. An important parameter determining the changes taking place in the deposited waste is the presence of petroleum pollutants, which age as a result of the course of many processes, including evaporation, dissolution, transport of water-soluble substances, and biodegradation. The neutralization of old petroleum pollutants in the areas of liquidated mines and in the areas of old waste pits is one of the key ecological problems faced by the oil and gas mining industry.

One of the main pollutants causing the inhibition of the development and growth of flora in these areas are petroleum hydrocarbons (TPH, BTEX, PAHs), the concentration of which in old soil varies widely and varies within wide ranges. Individual terrestrial ecosystems, constituting an ecological unit which includes groups of living organisms, form a certain whole adapted to the conditions in the biotope. Introducing petroleum pollutants to ecosystems disturbs the course of the natural cycles of matter and energy circulation. The stimulation of the development of some groups of microorganisms and the inhibition of the activity of the remaining ones are observed. In addition, petroleum pollution causes soil distortions, leading to the reduction of water capacity due to partial or complete filling of soil pores, impeding or preventing the exchange of air between the soil and the atmosphere. The excess of organic carbon in relation to nitrogen and phosphorus content causes an acute shortage of these components for microorganisms and plants, degradation of the physicochemical properties of soil colloids (sorption capacity, ion exchange), and causes visible and unfavorable changes in the pH of the environment [2,3].

The elimination of hazardous, toxic and mutagenic substances from the environment (soil) is one of the main goals of environmental engineering. There are many techniques for remediating soil contaminated with petroleum substances, including thermal (thermal combustion), physical-chemical (soil washing, solidification, vacuum desorption, photolysis, chemical degradation, electrokinetic method) and biological (biodegradation, phytoremediation) methods. One of the most frequently used methods of treatment of soil contaminated with petroleum substances is biodegradation. Biodegradation is a much more economical method (no need to use complicated apparatus), safe and environmentally friendly than conventional thermal and physico-chemical methods. It is characterized by high efficiency and a wide spectrum of activity thanks to the use of agricultural microorganisms to decompose various types of pollutants. On the other hand, not all chemicals can be removed from the environment by biodegradation, and the time of land remediation by biological methods may be longer than by thermal or physico-chemical methods. Biodegradation can be carried out *in-situ*, i.e., in the place of contamination, or *ex-situ*, i.e., outside the place where contamination occurs. The advantage of conducting the biodegradation process with the *in-situ* method is the possibility of cleaning land in urbanized areas, while the *ex-situ* method enables the intensification of the contaminated soil remediation processes by controlling the process conditions and removing leachate containing harmful substances. 

The biodegradation of petroleum hydrocarbons is a complex process depending on many factors that can be divided into 3 main groups: factors related to pollution (xenobiotic concentration [4,5], age of pollution [6,7], physico-chemical properties [6,8,9,10], symmetry and particle size [11,12]), environmental factors [4] (temperature [13,14,15], pH of the environment [4], the presence of oxygen [4,6,9,16,17,18], humidity [4,6], the presence of nutrients [4,18,19,20], properties of the soil matrix [6,17,21]) and biological factors (quantitative and qualitative composition of microorganisms, bioavailability of the pollutant for organisms supporting decomposition, spatial distribution, the adaptability of microorganisms, interactions between species and within the same species, enzymatic activity [4,22,23,24,25,26,27]). 

The ability of microorganisms to adapt to pollutants plays a particularly important role in the decomposition of xenobiotics. At the molecular level, adaptation is explained by the genetic variation of microorganisms (resulting from the presence of the relevant genes), and then the selection of those cells that have acquired the ability to use xenobiotics as a source of carbon and energy. Petroleum pollutants constitute a complex, multi-component system and differ in their susceptibility to microbial attack. The susceptibility of hydrocarbons to microbial degradation can be broadly classified as follows: linear alkanes > branched alkanes > monoaromatic hydrocarbons (BTEX) > cyclic alkanes > polycyclic aromatic hydrocarbons [28]. There are several hundred species of microorganisms capable of biodegradation of petroleum pollutants, but most of them can metabolize a limited range of hydrocarbons [4]. For this reason, for their biodegradation, it is advisable to use a mixture of microorganism cultures with an extensive enzyme apparatus [29,30]. It is preferable to prepare bacterial consortia—biopreparations based on autochthonous microorganisms previously isolated from the soil in order to avoid the antagonistic influence of the autochthonous soil microbiota on foreign cultures, which creates a high probability of survival in a contaminated biocenosis, and thus guaran—tees a sufficiently high biodegradation potential. The most commonly reported bacteria in the literature capable of degrading both aliphatic and aromatic hydrocarbons the following types: *Achromobacter* [31], *Acinetobacter* [32], *Bacillus* [33,34,35,36], *Dietzia* [37], *Gordonia* [38,39,40], *Micrococcus* [41,42], *Mycobacterium/Mycolicibacterium* [43,44,45,46,47,48,49,50,51], *Nocardia* [52,53], *Pseudomonas* [54,55,56,57,58], *Rhizobium* [59,60], *Rhodococcus* [44,61,62,63,64,65,66], *Sphingomonas* [17,67,68,69,70], *Stenotrophomonas* [71,72,73] and *Sphingobacterium* [34,74,75]. A combination of bioaugmentation treatments (inoculation of the contaminated soil-water environment with microorganisms characterized by high biodegradation efficiency of petroleum pollutants, including TPH, BTEX and PAHs) and biostimulation (introduction of oxygen, water, biogenic substances and surface-active substances to the soil-water environment in order to increase the activity of microorganisms autochthonous species) allows for a significant improvement in the efficiency of the biodegradation process of petroleum hydrocarbons.

Currently, more and more research is being carried out on accelerating the biodegradation process of petroleum pollutants through biotechnological processes with the use of a bacterial consortium (biopreparations) prepared on the basis of autochthonous bacteria and additives accelerating the decomposition of hydrocarbons. An interesting solution seems to be the use of a biopreparation in the inoculation process in combination with biopolimers, e.g., γ-PGA.

Biopolymers—polymers produced by living organisms, fulfilling specific functions in these organisms. A number of biopolymers have an industrial application; in the oil and gas industry, polysaccharide biopolymers such as starch, cellulose and guar gum and their derivatives are used, and to a lesser extent xanthan gum, scleroglucan and chitin/chitosan [76]. Biopolymers that are not polysaccharide compounds have not aroused interest so far. However, in recent years, the attention of a number of researchers has focused on a compound of a polyamide nature—γ-polyglutamic acid (γ-PGA) [77]. A characteristic feature of γ-PGA is that it is made of one type of monomer—glutamic acid. The monomers in γ-PGA are linked by an amide bond between the α-amino group of one monomer and the α-carboxyl group of the other. γ-PGA is a product of bacteria and archaea, and only a few species have the ability to produce it [78,79]. Particularly preferred is the genus Bacillus, because most of the γ-PGA-synthesizing organisms belong to this genus (*Bacillus amyloliquefaciens*, *Bacillus licheniformis*, *Bacillus megaterium*, *Bacillus subtilis*, as well as the highly pathogenic organism—*Bacillus anthracis*) [80]. Particular species and even strains produce γ-PGA with different molecular weights; from 100 kD to 5000 kD [81,82], which is reflected in its physical properties. The higher the molecular weight, the greater the density of the aqueous γ-PGA solution. Depending on the environmental conditions, γ-PGA can assume various conformational states, e.g., in lower pH it will be in the form of an α-helix, and in higher pH in the form of a random coil [83]. On the other hand, increased temperature causes the aggregation of γ-PGA molecules and the formation of fibrous structures [84]. PGA can be produced from a variety of precursors. In general, γ-PGA-synthesizing microorganisms fall into 2 groups. The first one is strictly dependent on the presence of L-glutamic acid in the medium, the second one can use various precursors, such as citric acid, glucose, α-ketoglutaric acid, glycerol and various amino acids, e.g., alanine, arginine, ornithine, proline and aspartic acid, in particular glutamine. By growing the bacteria from the above-mentioned compounds in various combinations, γ-PGAs of various molecular weights can be obtained and the efficiency of the process can be controlled [77]. 

The changing properties of γ-PGA (depending on the environment in which it is located) have attracted the attention of many researchers in terms of the industrial application of this biopolymer. Moreover, the compound has many advantages: it is highly soluble in water, non-toxic to living organisms even in very high concentrations, biodegradable (although mainly by organisms capable of synthesizing it), and even edible [85]. Therefore, it is currently used as a flocculant [86,87], an additive to fertilizers [88] and food [89]. Its presence in human food has been known for a relatively long time—traditional Japanese natto (based on fermented soybeans) contains a lot of γ-PGA, and now it is suggested that γ-PGA has a positive effect on lowering sugar levels, and therefore has a health-promoting effect [90]. Other features—biocompatibility and lack of immunogenicity—allow it to be used as a medical and cosmetic agent [91]. The use of γ-PGA in environmental engineering has also begun to be considered. Favorable results have been obtained in the application of γ-PGA in the remediation of soils contaminated with heavy metals [92,93,94]. γ-PGA has also been reported to have a positive effect on the removal of crude oil from marine sediments [95] and trichlorethylene (TCE) from the water bearing zone [96]. Therefore, we decided to experimentally check whether the presence of γ-PGA would support the decomposition of petroleum substances (TPH, BTEX, PAHs) in soils inoculated with a biopreparation developed on the basis of bacterial microorganisms.

We hypothesized that it was possible to increase the efficiency of biodegradation of petroleum pollutants by using a biopreparation based on autochthonous bacteria with the addition of γ-PGA in the inoculation process. The aim of the research was: (a) to determine the efficiency of the biodegradation process of petroleum pollutants (TPH, BTEX, PAHs) during inoculation with a biopreparation prepared on the basis of autochthonous bacteria isolated, multiplied and selected from the treated soil, carried out for a period of 6 months on a semi-technical scale using the *ex-situ* prism method, (b) determining the degree of increasing the efficiency of biodegradation of petroleum pollutants during the application of the PGA additive in the inoculation process as a function of the duration of biodegradation processes, (c) toxicological monitoring of soil as a function of time during soil biodegradation processes together with the sensitivity assessment of the biotests applied. The efficiency of biodegradation of petroleum substances was assessed on the basis of respirometric tests, chromatographic analyses and toxicological tests with the use of a new generation toxicological test set, the bioindicators of which belonged to different taxonomic groups (bacteria, crustaceans and plants).

## 2. Materials and Methods

### 2.1. Soil and Microorganisms

In the study of the biodegradation of petroleum substances, the AB soil (initial soil) collected from the waste pit G11 (N: 49°40′31.23′′, E: 22°04′30.86′′) in the area of the crude oil mine located in south-eastern Poland was used. This soil is characterized by a high concentration of petroleum pollutants, i.e., aliphatic hydrocarbons (19,774.23 mg/kg DM), monoaromatic hydrocarbons (17.35 mg/kg DM) and polycyclic aromatic hydrocarbons (27.03 mg/kg DM). The content (%) of individual components of petroleum pollutants in the soil is provided in the Supplementary Materials on Figure S1.

For soil inoculation, biopreparation A1 was used, developed on the basis of bacterial strains (came from the hydrocarbon-degrading microbial collection of the Department of Microbiology at the Oil and Gas Institute–National Research Institute, Krakow, Poland), isolated formerly from the AB soil: *Dietzia* sp. *IN133*, *Gordonia* sp. *IN138 Mycolicibacterium frederiksbergense IN53*, *Rhodococcus erythropolis IN119*, *Rhodococcus* sp. *IN136* and *Pseudomonas* sp. *IN132.* A commercially available preparation, Ambiogel^®^ (Ambioteco, Staszów, Poland), containing 10% (*w/w*) pure gamma-polyglutamic acid, was used as a γ-PGA additive. Table 1 presents the species affiliation of bacterial strains occurring in the biopreparation A1.

All reagents used for the cultivation of microorganisms came from Avantor Performance Materials Poland S.A. (former POCH Gliwice) (Gliwice, Poland) and were of analytical purity with the exception of nutrient broth which were purchased from Biomaxima S.A. (Lublin, Poland).

### 2.2. Biopreparation Development

The stains were grown separately in nutrient broth supplemented with the addition of sodium acetate (0.2%, *w/v*) and incubated at 25 °C at 150 rpm for 72 h to obtain a cell number of 10^8^–10^9^ cfu/cm^3^. The biopreparation used for further research was prepared by mixing equal volumes of all strains. The ability to use crude oil and various hydrocarbons as the sole source of carbon and energy was tested by incubating each of the strains in a mineral medium with the addition of particular compounds. The test medium was described in the study of Brzeszcz et al., 2020 [97,98]. Simultaneously, tests were carried out according to the method described by Wrenn and Venos (1986) [99]. The characteristics of the individual strains were established on the basis of microscopic observations, colony morphology, growth on the selective agar media and biochemical profiles (API Coryne and 32GN bioMerieux tests). The strains were identified phylogenetically by sequencing the 16S rRNA gene as previously described [100]. The obtained sequences were deposited in the NCBI database, and their accession numbers together with the properties of the strains are presented in Table 2.

### 2.3. Biodegradation Experiments

**Figure 1:**
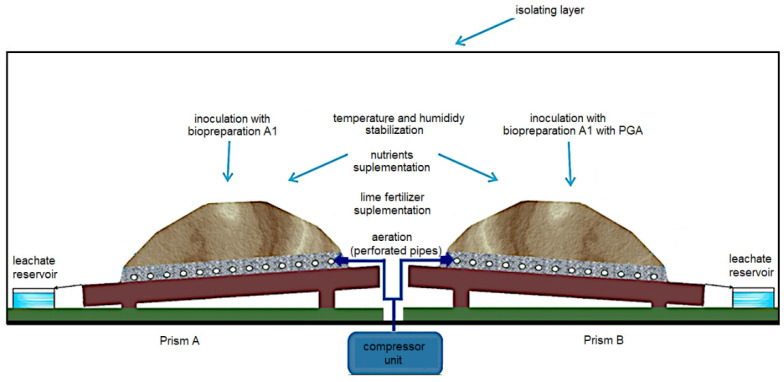
Stand for testing the process of biodegradation of petroleum hydrocarbons.

A workstation for tests carried out on a semi-technical scale in the conditions of *ex-situ* treatment of soil contaminated with petroleum substances was created in the Oil and Gas Institute—National Research Institute, Krosno Division. The structure of the test workstation was as follows: a foil was placed directly on the floor to protect the surface against the spread of contamination of the room, for which a gravel bed of appropriate granulation was used as the first layer. Perforated pipes were laid on the gravel layer, through which compressed air was supplied from the compressor unit. Then the pipes were covered with another layer of gravel, secured with a technical fabric to prevent clogging. On the base prepared in this way, two identical prisms of AB soil were placed in the amount of 30 kg (per 1 prism) (Figure 1).

Before the soil inoculation process, the proper C:N:P ratio was ensured, which should be at the level of 100:10:1 [36,44,100,101] in order to ensure optimal conditions for the proper activity of microorganisms contained in the biopreparation. This was carried out by supplementing nitrogen and phosphorus with the Azofoska mineral fertilizer with the following composition: 13.6% total N, 8.1% ammoniacal nitrogen, 5.5% nitric nitrogen, 6.4% soluble P_2_O_5_, 19.1% K_2_O in the form of K_2_SO_4_, 4.4% MgO in the form of soluble MgSO_4,_ and microelements (0.17% Fe, 0.27% Mn, 0.18% Cu, 0.045% Zn, 0.09% Mo). Nutrients (nitrogen and phosphorous–present in the soil as ions: NH^4+^-N, NO_3_-N, PO_4_-P) were determined using the Perkin Elmer Lambda 40 spectrophotometer (Waltham, MA, USA). The soil pH was corrected by supplementing lime in the amount of 1.0–1.5 g/kg of soil until the optimum pH of 7.5–7.6 was obtained [44]. The prepared AB soil was inoculated with biopreparation A1 (2 dm^3^)–prism A, or biopreparation A1 (2 dm^2^) with the addition of γ-PGA (2 dm^3^ solution Ambiogel^®^—hereinafter referred to as γ-PGA)—prism B. The prisms were covered with a foil tunnel, which allows to maintain a constant temperature inside at the level of approx. 20–25 °C, achievable in field conditions in the summer season of a moderate climatic zone and with constant humidity (20–25%). During the treatment process, the content of TPH, BTEX and PAHs in the prism inoculated with the biopreparation A1 (Soil A2, A4, A6) and with the biopreparation A1 with γ-PGA (Soil B2, B4, B6) was monitored, and changes in the level of soil toxicity were determined using the toxicological test package. The process of biodegradation of petroleum hydrocarbons in ex-situ conditions was carried out for 180 days.

### 2.4. Respirometric Tests

The respirometric tests consisted in measuring the biological activity in the analyzed soil under aerobic conditions. The research was carried out with the use of the Oxi-Top set (WTW, Weilheim, Germany). Samples weighing 30 g were placed in measuring vessels to which biopreparation A1 (2 cm^3^) with a density of 109 cfu mL^−^^1^ and biopreparation A1 (2 cm^3^) with γ-PGA (solution γ-PGA 1 and 2 cm^3^) were added. Finally, the humidity of each system was adjusted to 25% by adding distilled water. At the same time, reference samples were prepared in which the analysed soil and inoculant solutions were placed separately. Then all measuring vessels were closed with OxiTop measuring heads, then placed in an incubator and thermostated at 20 °C for 60 days. The Oxi-Top Control system measuring heads read the pressure values in the closed system every two hours. The collected data was transferred via the IR interface to the Oxi-Top OC 110 controller, where it could be processed graphically and statistically using the Achat OC program. The conversion of the measured pressure into the value of consumed oxygen ($m_{O_{2}}$) is carried out according to the Formula (1): At the end of the measurement, the data was sent to a PC, where, using Excel, curves of the dependence of the amount of oxygen consumed [mg/dm^3^] and the duration of the experiment [days] were prepared. All experiments were performed in triplicate with five replications for each treatment [44,102,103,104].

(1) $$m_{O_{2}}=\frac{M\left(O_{2}\right)}{R\times T_{m}}\left(V_{g}+\alpha \frac{T_{m}}{T_{0}}\right)\times \Delta p$$

where: *M* (*O*_2_)—molar mass of oxygen [kg/mol], *V_g_*—volume of free gas [m^3^], *R*—gas constant [J·mol^−^^1^·K^−^^1^], *T_m_*—temperature measurement value [K], *T*_0_—reference temperature (273.15 K), *α*—absorption coefficient (0.03103), Δ*p*—pressure drop in the test [Pa].

### 2.5. TPH Extraction and Quantification 

Isolation of TPH from the soil matrix was carried out using the ultrasound-assisted solvent extraction method. For this purpose, 10 g of air-dry soil sample was placed in an Erlemnajer flask and extracted with 3 portions of solvent (dichloromethane, Avantor Performance Materials Poland S.A., Gliwice, Poland). The soil extraction time was 15 min. for each portion of the solvent. Extraction was carried out in a Sonoswiss SW 6H ultrasonic bath (Sonoswiss AG, Ramsen, Switzerland) with an ultrasound frequency of 30 kHz [44,105]. The obtained extracts were purified by SPE solid phase extraction on Bakerbond No. 7213-03 columns packed with florisil (J.T.Baker^®^, Phillipsburg, NJ, USA). The obtained extracts were concentrated on a ChemLand RE100-Pro rotary vacuum (ChemLand, Stargard Szczeciński, Poland evaporator to a volume of 1 cm^3^. The efficiency of the ultrasonically assisted solvent extraction was estimated to be 95.9% using the o-terphenyl replacement standard [44,98].

The TPH analysis in soil samples was performed on a Perkin Elmer Clarus 500 GC chromatograph (Waltham, MA, USA) equipped with a flame ionization detector (FID), with the following device parameters: RTX-1 capillary column of fused silica, 30 m × 0.53 mm (Restek, Bellefonte, PA, USA), injector temperature = 290 °C, FID detector temperature = 320 °C, oven temperature programme: 30 °C (2 min. isothermal run), 30–105 °C (temperature increase 10 °C min^−1^), 105–285 °C (temperature increase 5 °C min^−1^), and 285 °C (5 min isothermal run), carrier gas (He) flow 20 mL min^−1^ [44]. The total TPH content in soil samples was assessed on the basis of the certified BAM K010 standard (Tusnovic Instruments, Krakow, Poland). Standard mixture paraffin hydrocarbons: (nC_6_–nC_44_) ASTM^®^ D2807 (Supelco, Saint Louis, MO, USA) and certified standard mixture Fuel Oil Degradation Mix n-C_17_, pristane, n-C_18_, phytane No A029668: (Restek, Bellefonte, PA, USA) were used to quantitatively the individual n-alkanes included in the petroleum pollutants. A certified standard C_30_ 17α(H), 21β(H)-hopane No 08189 (Sigma-Aldrich, Saint Louis, MO, USA) was used as a biomarker [44].

### 2.6. BTEX Extraction and Quantification

Headspace gas chromatography was used to determine the content of monoaromatic hydrocarbons (BTEX) in soil [106]. A soil sample with a mass of 6.5 g (as calculated for the dry mass of soil, approx. 5 g) was placed in an ampoule, which was tightly closed with a capping machine and inserted into an autosampler. Before the analysis, the sample was thermostated for 10 min. at 90 °C. The ampoule was filled with a carrier gas (helium) for 3 min, and then a sample of gaseous analytes desorbed to the headspace was collected using a dosing needle heated to the temperature of 95 °C, which was sent via a transfer line (temperature 100 °C) to the injector of the Clarus 500 gas chromatograph with an FID detector, connected to HeadSpace TurboMatrix 16 (Perkin Elmer Inc., Waltham, MA, USA). The injection time was 0.04 min. The operating conditions of the chromatograph were as follows: fused silica capillary column (RT-TCEP: 60 m × 0.25 µm) (Restek, Bellefonte, PA, USA), using the following temperature parameters: injector temperature = 200 °C, FID detector temperature = 280 °C, TCD detector temperature = 150 °C, furnace temperature program: 60 °C—isothermal course for 5 min, 60–100 °C—temperature increase at a rate of 5 °C/min, 100 °C—isothermal course for 10 min. To quantify the total BTEX content, the certified standards of Restek No. 30051 (Restek, Bellefonte, PA, USA) and Supelco No. CRM48026 (Supelco, Saint Louis, MO, USA) [106] were used.

### 2.7. PAHs Extraction and Quantification

The isolation of analytes from the soil matrix was carried out using the QuEChERS method (Quick, Easy, Cheap, Effective, Rugged, Safe). For this purpose, 5 g of air-dry soil was weighed into a 50 mL vial, and then 5 cm^3^ of distilled water and 10 cm^3^ of acetonitrile (Avantor Performance Materials Poland S.A., Gliwice, Poland) were added to it. The contents of the sachet with the MgSO_4_ + NaCl extraction mixture No. SST640 (Interchim, Montluçon, France) were then added to the vial. The samples prepared in this way were shaken for 5 min, and then centrifuged for 10 min at a speed of 3500 rpm [107].

Purification of the extract was performed using the dSPE method using purification vials filled with MgSO_4_ and PSA No. JO3937 (Interchim, Montluçon, France). 1 cm^3^ of the soil extract was transferred to a purification vial, which was then shaken for 5 min and centrifuged for 10 min at 8000 rpm. The obtained extracts were filtered through a syringe filter with a pore diameter of 25 µm and subjected to chromatographic analysis [107]. 

The analysis of polycyclic aromatic hydrocarbons (PAHs) in soil samples was performed on a Vanquish Core high pressure liquid chromatograph (HPLC) with the following device parameters: NUCLEODUR C_18_ PAH column, 125 mm × 4 mm, 3 μm (Marcherey-Nagel, Germany), detector: UV-VIS and fluorimetric (FLD) dispenser: automatic, dispensing volume: 10 μL, eluents: A-methanol (Avantor Performance Materials Poland S.A., Gliwice, Poland) 70%, B-acetonitrile (Avantor Performance Materials Poland S.A., Gliwice, Poland), flow: 1.5 mL/min, gradient: 20% B for 1.5 min, 20–50% B in 1.5 min, 50–100% B in 1 min, 100% B for 1 min, 100–0% B for 3 min, 100% A for 3 min. Certified PAH-Mix solution ref. 722393 (Marcherey-Nagel, Düren, Germany) was used to quantify the individual PAHs included in the petroleum pollutants. 

### 2.8. Ecotoxicological Analyses

To evaluate the effectiveness of the biodegradation process of soils contaminated with petroleum substances, a set of toxicological tests used in bioindication analyses was applied. Bioindication analyses are one of the newest methods of environmental toxicity studies in which lethal and sub-lethal effects (morphological changes, diseases) of indicator organisms are observed. During the biodegradation of petroleum hydrocarbons as a result of chemical and microbiological transformations, metabolites of various biological activity may be produced. Toxicological tests quickly provide information on the toxic effects of chemical compounds (both acute and chronic) on a selected indicator organism or population. In this publication, 4 toxicological tests were used to evaluate the effectiveness of the remediation treatments used in the process of soil remediation in ex-situ conditions: Phytotoxkit^TM^ (MicroBioTests Inc., Nazareth, Belgium), Ostracodtoxkit ^TM^ (MicroBioTests Inc., Nazareth, Belgium), Microtox^®^ Solid Phase Test (SDI, Newark, DE, USA) and Ames (Muta-ChromoPlate^TM^ Kit (EBPI, Mississauga, ON, Canada) [44]. The list of toxicological tests used in the research along with the description of the procedures for their performance is presented in Supplementary Materials.

### 2.9. Mathematical Model of TPH, BTEX and PAHs Biodegradation

In order to work a simplified mathematical model of the biodegradation of petroleum hydrocarbons during *ex-situ* method to normalize the concentrations of TPH analytes (Σ n-C_10_–n-C_22_ and Σ n-C_23_–n-C_40_), BTEX (benzene, toluene, ethylbenzene, xylenes) and PAHs (2-ring PAHs, 3-ring PAHs, 4-ring PAHs, 5-ring PAHs, 6-ring PAHs) the biomarker C3017α (H), 21β (H)-hopan (Supelco, Saint Louis, MO, USA) was used. The biomarker used in biodegradation tests allows for the elimination of analytical errors that may occur during the determination of the analyzed groups of analytes with the chromatographic method. The standardized results of chromatographic determinations performed during biodegradation were used to develop a primary mathematical model describing its course, which was described by Equation (2) [44,100].

C/Cx = (C/Cx)0 exp (−kt) (2)

where: C—analyte concentration, Cx—biomarker (Hopane) concentration, k—first-order rate constant [d^−1^], (C/Cx)0—normalized analyte concentration at the starting point, and t—process duration [d].

The nonlinear regression analysis with the use of Equation (2) made it possible to determine the first-order biodegradation constant (k) and the correlation coefficient (r^2^), which determined the fitting of the measuring points to the theoretical curves of the TPH, BTEX and PAHs biodegradation process.

### 2.10. Data Analysis and Statistical Information

Statistical analysis was performed with Statistica 14.0 (TIBCO Software Inc., Palo Alto, CA, USA). Standard deviation (SD), standard deviation (RSD) (%) and Pearson correlation coefficient were calculated. All obtained data were previously tested for normal distribution. They were then checked by a one-way ANOVA test. In the case of obtaining significant results in the ANOVA test, Tukey’s pairs post-hoc test was performed. The significance was set at *p* < 0.05 [44].

## 3. Results and Discussion

### 3.1. Respirometric Tests

**Figure 2:**
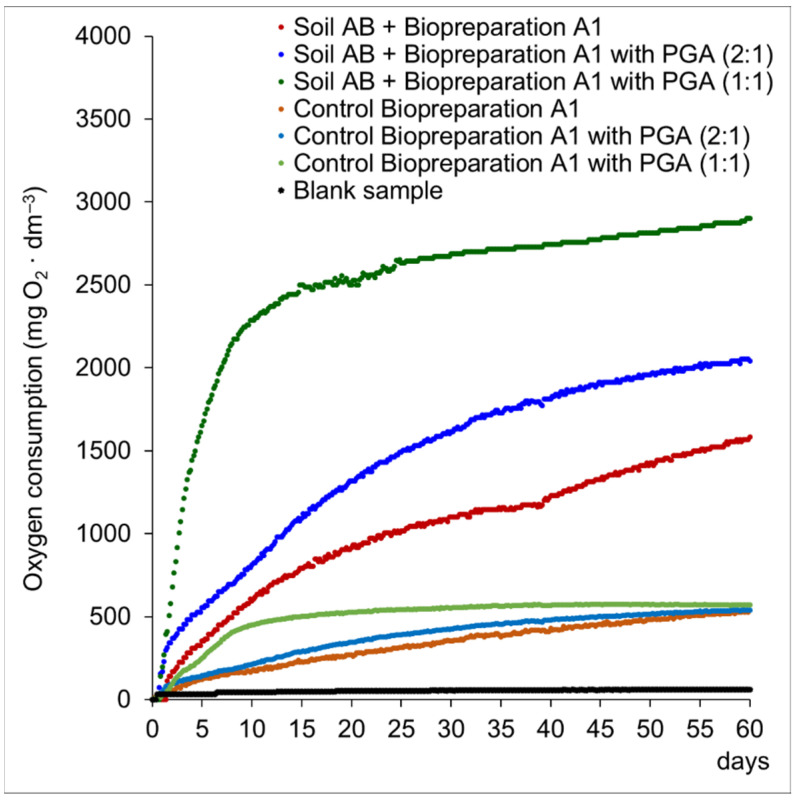
Dependence of the amount of oxygen consumed [mg·dm^−3^] on time in AB soil inoculated with biopreparation A1 and biopreparation A1 with γ-PGA addition. Control-sterile uncontaminated “pure” soil inoculated with biopreparation A1 and biopreparation A1 with γ-PGA. Blank sample is soil AB with distilled water.

Respirometric tests using the OxiTop kit are based on highly accurate, automatic pressure measurements using a piezoresistive electronic pressure sensor (OxiTop Control) in a sealed bottle at a constant temperature. The conducted respirometric tests on control samples (sterile soil not contaminated with hydrocarbons) showed the biological activity of biopreparation A1 and biopreparation A1 with the addition of PGA after 60 days of the test at the level of 526 O_2_/dm^3^ and 536 O_2_/dm^3^ (volume ratio of biopreparation A1 and γ-PGA solution was 2:1) and 570 O_2_/dm^3^ (volume ratio of biopreparation A1 and γ-PGA solution was 1:1), respectively. The oxygen consumption in the AB soil sample, which was not subjected to the inoculation process (Blank sample) after the end of the test, was 62.1 O_2_/dm^3^. After 60 days of the test, the oxygen consumption in the soil sample inoculated with biopreparation A1 was 1582 mg O_2_/dm^3^, while in the sample inoculated with biopreparation A1 with the addition of γ-PGA 2040 O_2_/dm^3^ (volume ratio of biopreparation A1 and γ-PGA solution was 2:1) and 3769 mg O_2_/dm^3^ (volume ratio of the solutions of biopreparation A1 and γ-PGA solution was 1:1) (Figure 2). The increase in microbiological activity in the reaction environment proves that the microorganisms use carbon contained in aliphatic hydrocarbons, monoaromatic hydrocarbons and polycyclic aromatic hydrocarbons as a food source. The result of the increased biological activity is the increase in oxygen consumption by the examined bacterial consortia over time, which results in the biodegradation of petroleum substances. The obtained results of respirometric tests prove the effectiveness of the biodegradation process of petroleum hydrocarbons with the use of inoculants in the form of biopreparation A1 and biopreparation A1 with the addition of γ-PGA. Based on the conducted respirometric tests, the method of AB soil inoculation with the use of A1 biopreparation A1 and biopreparation A1 with the addition of γ-PGA in the volume ratio of 1:1 solution was selected for further studies of the biodegradation process of petroleum hydrocarbons carried out by the *ex-situ* prism method.

### 3.2. Assessment of Biodegradation of Petroleum Pollutants on the Basis of Chromatographic Analyses

The effectiveness of the biodegradation process of petroleum hydrocarbons in soils inoculated with the biopreparation A1 and the biopreparation A1 with γ-PGA was determined on the basis of chromatographic analyses. 

#### Chromatographic Analysis of TPH, BTEX and PAHs

As a result of inoculation of soil AB with the biopreparation A1, after 6 months of the purification process, the TPH content was reduced from 19,774.23 mg/kg DM to 6717.33 mg/kg DM, while as a result of inoculation with the biopreparation A1 with the addition of γ-PGA, the TPH content was reduced to 4111.47 mg/kg DM. 

The course of TPH biodegradation in the AB soil inoculated with the biopreparation A1, as well as with the biopreparation A1 with the addition of γ-PGA, was similar in the following months of the process, but its pace decreased, and at the end remained at a similar level. Degrees of biodegradation of TPH content during the purification process by inoculation with the biopreparation A1 were: after 2 months 35.03% (Soil A2), after 4 months 53.18% (Soil A4), and after 6 months 66.03% (Soil A6). The degree of TPH reduction during inoculation with the biopreparation A1 with the addition of PGA was higher and was as follows: after 2 months 48.21% (Soil B2), after 4 months 65.73% (Soil B4), and after 6 months 79.21% (Soil B6).

The results of the chromatographic analyses showed that as a result of inoculation with both the biopreparation A1 and the biopreparation A1 with the addition of γ-PGA, the biodegradation of aliphatic hydrocarbons with a carbon chain length of n-C_9_–n-C_21_ was the fastest. The degrees of biodegradation of these hydrocarbons in soil after 2, 4 and 6 months were in the range of: 45.60–75.14 (Soil A2), 67.25–87.85 (Soil A4), 86.52–94.59% (Soil A6) and 56.87–85.49% (Soil B2), 88.46–96.67% (Soil B4) and 96.57–99.55% (Soil B6). Hydrocarbons in the n-C_22_–n-C_30_ range were also satisfactorily biodegradable within the range: Soil A2 (20.87–39.66%), Soil A4 (29.08–64.26%), Soil A6 (44.62–77.77%), Soil B2 (34.43–49.46%), Soil B4 (56.24–79.15%), Soil B6 (80.35–88.35%). Hydrocarbons with more than 30 carbon atoms in the molecule turned out to be the most difficult to biodegrade. The degrees of their biodegradation after 6 months of the treatment process in soil A6 were within the range of 25.13–40.60%, while in soil B6 it was within the range of 46.43–65.73%. The content of unidentified hydrocarbons in Soil A6 decreased by 59.49%, and in Soil B6 by 71.84%.

Chromatographic analysis allowed for a broader look at the course of the process, as it made it possible to determine the biodegradability of individual groups of hydrocarbons. Both in the soil inoculated with the biopreparation A1 and the biopreparation A1 with the addition of γ-PGA, the rate of removal of individual groups of hydrocarbons was in a decreasing order: n-C_10_–n-C_22_ > n-C_23_–n-C_28_ > n-C_28_–n-C_40_. This removal model is likely related to the chemical structure of the alkanes. Alkanes with a chain length of n-C_10_–n-C_21_ are the substances most willingly used by bacteria in metabolic processes [36,100,101,108], while alkanes with longer carbon chains are less biodegradable. Straight and short carbon chain hydrocarbons (n-C_10_–n-C_22_) present in soil are therefore perceived as the most attractive source of energy by any organism capable of degrading them. The ability of individual bacterial strains (present in the biopreparation) to decompose hydrocarbons also plays an important role in the process of biodegradation of aliphatic hydrocarbons. Bacterial strains capable of decomposing various types of petroleum hydrocarbons were used in the biopreparation A1. Nevertheless, each bacterial strain may prefer a different type of hydrocarbon. For instance, some bacteria can metabolize specific alkanes, while others break down aromatic hydrocarbons. Taking into account the ability of various bacterial strains to degrade aliphatic hydrocarbons, it should be noted that Dietzia e a broad spectrum of the ability to degrade oil-derived hydrocarbons in the n-C_6_–n-C_40_ range) [37]. *Pseudomonas* most effectively degraded n-alkanes in the n-C_14_–n-C_30_ range, [109], while *Rhodoccocus* in the n-C_13_–n-C_17_ range [63]. *Gorgonia* had the ability to effectively degrade both linear and branched aliphatic hydrocarbons [38]. 

**Figure 3:**
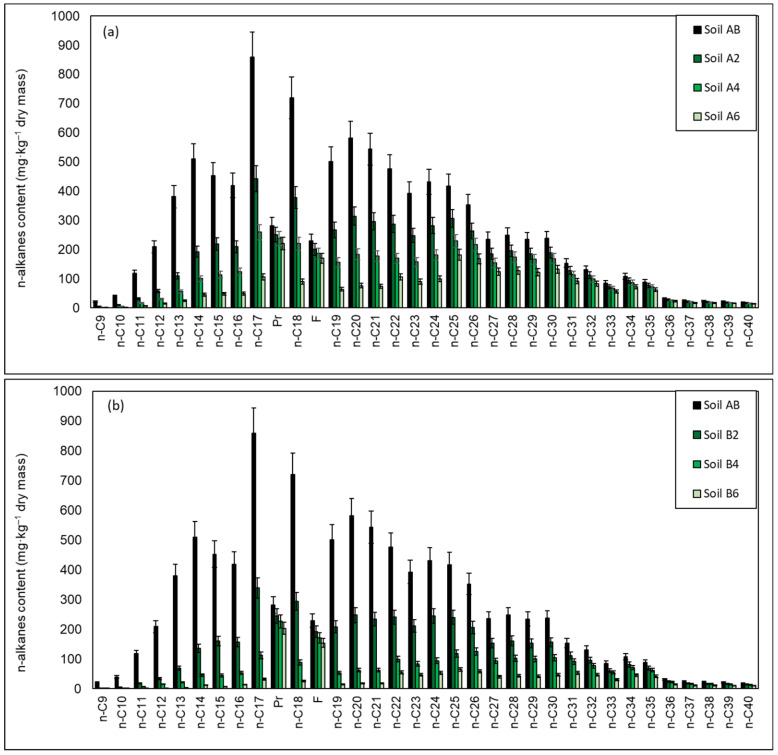
Residual content of n-alkanes in soil contaminated petroleum hydrocarbons inoculated (**a**) biopreparation A1, (**b**) biopreparation A1 with the addition of γ-PGA after 2 (Soil A2/Soil B2), 4 (Soil A4/Soil B4) and 6 (Soil A6/Soil B6) months of the biodegradation process carried out in ex-situ conditions. Control was non-inoculated Soil AB. (repetition number *n* = 7–10, *p* < 0.05).

A comparison of the content of n-alkanes in the tested soil (Soil AB) inoculated with (a) biopreparation A1; (b) biopreparation A1 with the addition of γ-PGA after 2 (Soil A2/Soil B2), 4 (Soil A4/Soil B4) and 6 (Soil A6/Soil B6) months of the biodegradation process is presented in Figure 3.

The indicators of biodegradation degree prove that the course of biological decomposition of n-alkanes is satisfactory, both in soil inoculated with the biopreparation A1 and in the biopreparation A1 with the addition of γ-PGA. In A6 soil these indicators decreased: n-C17/Pr from 3.0 to 0.48, n-C18/Ph from 3.14 to 0.53, while in B6 soil n-C17/Pr from 3.0 to 0.20, n-C18/Ph from 3.14 to 0.21. The presented results prove that with proper control of treatment processes (appropriate temperature, humidity, nutrient content, aeration) and extensive monitoring (chromatographic analysis), a satisfactory biodegradation efficiency of aliphatic hydrocarbons can be obtained. 

The chromatographic analyses performed showed that the addition of γ-PGA to the biopreparation increases the efficiency of biodegradation of aliphatic hydrocarbons. The comparative analysis carried out on identical samples of the initial soil AB inoculated with the biopreparation A1 and the biopreparation A1 with the addition of γ-PGA showed that the degree of TPH biodegradation after 6 months of the process in B6 soil is 13.18% higher than in A6 soil. 

As a result of the biodegradation process carried out under semi-technical conditions (*ex-situ* method) using the biopreparation as an inoculant, after 6 months of the process, the BTEX content was reduced from 17.45 mg/kg DM to 3.48 mg/kg DM. The results of the chromatographic analyses showed that during the inoculation with the biopreparation A1, the biodegradation of toluene took place the fastest and the degrees of its reduction after 2, 4 and 6 months were, respectively: 63.22%, 84.17% and 96.54%. Ethylbenzene and benzene turned out to be slightly less biodegradable, their biodegradation rates were, respectively, 60.47% and 59.17 in Soil A2, 80.08% and 79.98% in Soil A4, and 93.15% and 91.75% in Soil A6. The least biodegradable were xylenes, whose biodegradation rates after the end of the process were at the level of 39.91–46.85% (Soil A2), 55.73–62.29% (Soil A4), and 71.06–78.69% (Soil A6).

Biodegradation of monoaromatic hydrocarbons in the soil inoculated with the biopreparation A1 with the addition of γ-PGA was more effective, as evidenced by the reduction in BTEX content after 6 months of purification to the level of 1.71 mg/kg DM (90.19%). Compared to the soil inoculated with the biopreparation A1, the application of γ-PGA additive increased the degree of BTEX biodegradation by 10.11%. Toluene biodegradation rates over 6 months were 66.06% in Soil B2, 89.88% in Soil B4, and 98.89% in Soil B6. The decrease in the content of ethylbenzene and benzene after 2 months of the experiment was 65.93% and 63.89%, after 4 months it was 86.66% and 84.17%, and after 6 months it was 97.69% and 95.89%. The degrees of biodegradation of the least biodegradable xylenes were within the range of 45.51–50.33% (Soil B2), 66.49–71.19% (Soil B4) and 85.14–89.88% (Soil B6).

In both inoculation methods, the removal rate of particular monoaromatic hydrocarbons decreased in the order of toluene > ethylbenzene > benzene > p-xylene > m-xylene > o-xylene. The available literature data indicate that the degradation rate of particular hydrocarbons from the BTEX group is related to the type of bacterial strain used in the study [110,111]. Research by other scientists shows that the ability to degrade monoaromatic hydrocarbons for *Pseudomonas putida* and *Bacillus cereus* strains decreases in the following order: toluene > ethylbenzene > benzene > xylenes [111], while the data obtained by Shima indicate that for the *Pseudomonas putida* strain this ability decreases in the order toluene > benzene > xylenes > ethylbenzene. In the differences in the order of degradation of individual monoaromatic hydrocarbons, the authors refer to different environmental conditions from which given bacterial strains were isolated [111]. The degradation of BTEX by the *Rhodococcus* sp. decreases in the order of toluene > xylenes > ethylbenzene [112]. By contrast, *Bacillus amyloliquefaciens* degraded monoaromatic hydrocarbons in the order toluene > benzene, ethylbenzene, p-xylene > m-xylene > o-xylene [110]. In the case of inoculation with biopreparations, the rate of removal of individual BTEX by biodegradation may therefore be different depending on the qualitative and quantitative composition of the biopreparation. The use of a mixed culture to inoculate soil contaminated with petroleum hydrocarbons influenced the effectiveness of the BTEX biodegradation process, because interspecies interactions may be necessary for complete biodegradation of a multicomponent mixture of hydrocarbons [111,113].

Moreover, hydrocarbons from the BTEX group belong to volatile organic pollutants, which additionally affects the value of the determination of these compounds in soil samples collected after 2, 4 and 6 months of biodegradation under semi-technical conditions. The use of the aeration system in the construction of the stand to conduct the biodegradation process using the *ex-situ* prism method influenced the speed and efficiency of removing volatile organic pollutants (BTEX) from the soil [114]. The combination of inoculants capable of effectively removing hydrocarbons from the BTEX group and appropriate conditions (constant temperature 20–25 °C, aeration) for the biodegradation process allowed to achieve very high levels of reduction of these compounds after the end of the process (71.06–98.89%).

**Figure 4:**
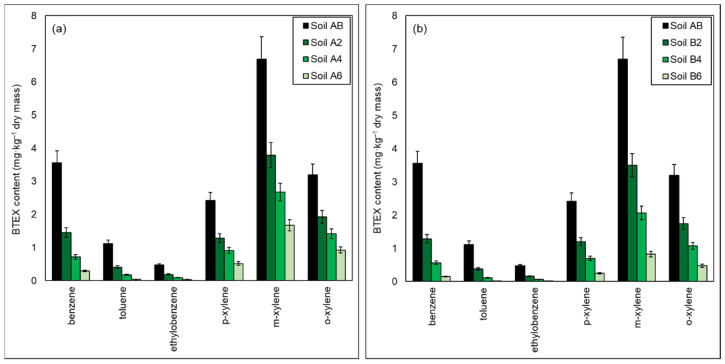
Residual content of BTEX in soil contaminated petroleum hydrocarbons inoculated (**a**) biopreparation A1, (**b**) biopreparation A1 with the addition of γ-PGA after 2 (Soil A2/Soil B2), 4 (Soil A4/Soil B4) and 6 (Soil A6/Soil B6) months of the biodegradation process carried out in ex-situ conditions. Control was non-inoculated Soil AB. (repetition number *n* = 7–10, *p* < 0.05).

A comparison of the monoaromatic hydrocarbons content in the tested soil (Soil AB) inoculated with (a) biopreparation A1; (b) biopreparation A1 with the addition of γ-PGA after 2 (Soil A2/Soil B2), 4 (Soil A4/Soil B4) and 6 (Soil A6/Soil B6) months of the biodegradation process is presented in Figure 4.

The performed chromatographic analysis allowed to determine the degree of reduction of particular identified PAHs. In the soil inoculated with the biopreparation A1, the highest degree of reduction of the content after the end of the biodegradation process was recorded for naphthalene and it was 49.72% after 2 months, 62.11% after 4 months, and 71.58% after 6 months. A slightly lower degree of biodegradation was obtained for 3-ring PAHs (ACE, FLU, PHEN, ANTH) 31.37–44.44% (Soil A2), 50.00–56.32% (Soil A4), and 63.73–64.44% (Soil A6). In the case of 4-ring PAHs (FLTH, PYR, B[a]A, CHRY), the reduction in their content was much lower and ranged from 14.06% to 30.14% after 2 months, from 23.47% to 38.93% after 4 months, and from 32.33% to 42.78% after 6 months. 5-ring PAHs and 6-ring PAHs were much more difficult to biodegrade, because the efficiency of the biodegradation process after 2 months of the process was determined at the level of 13.98–15.38%, after 4 months in the range of 22.68–20.78%, and after 6 months within the range of 24.62–30.06%. As a result of the biodegradation process carried out in semi-technical conditions (using the *ex-situ* method) with the use of a biopreparation A1, after 6 months of the process, the PAHs content was reduced from 27.03 mg/kg DM to 16.52 mg/kg DM, which is 38.86%. In turn, as a result of inoculation with a biopreparation A1 with the addition of γ-PGA, it was reduced to 13.19 mg/kg DM (51.18%). Therefore, the use of the γ-PGA addition to the biopreparation A1 increased the efficiency of the PAHs biodegradation process by 12.32%. Inoculation with the biopreparation A1 with the addition of γ-PGA allowed to reduce the content of naphthalene after 2 months by 59.93%, after 4 months by 77.41%, and finally after 6 months by 81.06%. Reduction levels of 3-ring PAHs (ACE, FLU, PHEN, ANTH) were at a level of 45.10–52.22% in Soil B2, 60.78–63.33% in Soil B4 and 68.63–72.22% in Soil B6, while 4-ring PAHs (FLTH, PYR, B[a]A, CHRY) at a level of 26.93–37.05% in Soil B2, 39.11–46.73% in Soil B4 and 44.65–56, 24% in Soil B6. A slightly lower degree of reduction, within the range from 22.37% to 28.87% in Soil B2, from 31.71% to 33.00% in Soil B4 and from 40.30% to 43.17% in Soil B6, was obtained for 5-ring PAHs (B[b]F, B[k]F, B[a]P). The degree of biodegradation of 6-ring PAHs (D[ah]A, B[ghi]P, IND) was the lowest among all PAHs, as it was 15.11–25.40% after 2 months, 25.72–34.72% after 4 months and 6 months after 35.48–37.83%. 

PAHs can be adsorbed, volatilized, photolyzed and chemically degraded, but nevertheless microbiological degradation is the main process of their removal from the environment. The degradation of PAHs depends on the environmental conditions, the number and type of microorganisms, the nature and chemical structure of the degraded chemical compound [115]. In general, the biodegradation rate of PAHs decreases with an increase in the number of aromatic rings in the molecule 2-ring PAHs > 3-ring PAHs > 4-ring PAHs > 5-ring PAHs > 6-ring PAHs [4]. 2-ring naphthalene is relatively volatile, soluble and biodegradable. Aromatic hydrocarbons with more rings in the molecule are less biodegradable and less soluble in water. This is because the stability of polycyclic aromatic hydrocarbons increases with increasing molecular weight. Moreover, as the number of aromatic rings in the molecule increases, the vapor pressure of PAHs and their solubility in water decrease, while the resistance to oxidation and reduction increases [12]. It is known that many species of bacteria are capable of decomposing PAHs, and most of them are isolated from contaminated soil. Strains of *Acinetobacter*, *Arthrobacter*, *Burkholderia*, *Dietzia*, *Mycobacterium*/*Mycolicibacterium*, *Rhodococcus Pseudomonas* and *Sphingomonas* are among the commonly known PAHs degraders [115]. The use of A1 biopreparation developed on the basis of indigenous bacteria capable of decomposing a wide range of petroleum pollutants, including PAHs allowed for an effective reduction of the analyzed polycyclic aromatic hydrocarbons in the studied soil. The use of the γ-PGA additive allowed to increase the efficiency of the PAHs biodegradation process, compared to the A1 biopreparation. 

**Figure 5:**
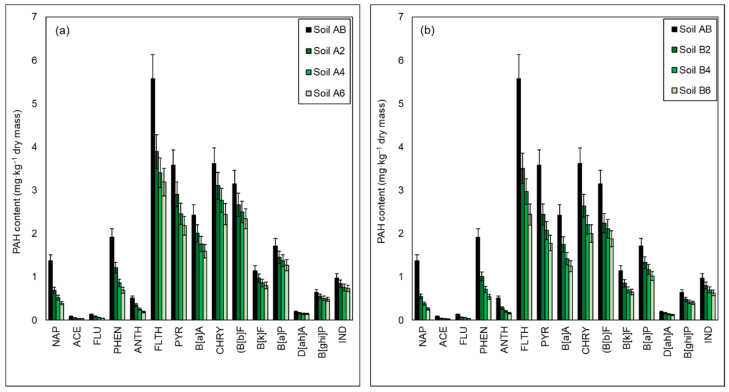
Residual content of PAHs in soil contaminated petroleum hydrocarbons inoculated (**a**) biopreparation A1, (**b**) biopreparation A1 with the addition of γ-PGA after 2 (Soil A2/Soil B2), 4 (Soil A4/Soil B4) and 6 (Soil A6/Soil B6) months of the biodegradation process carried out in ex-situ conditions. Control was non-inoculated Soil AB. (repetition number *n* = 7–10, *p* < 0.05). (naphthalene (NAP), acenaphthene (ACE), fluorene (FLU), phenanthrene (PHEN), anthracene (ANTH), fluoranthene (FLTH), pyrene (PYR), benzo[a]anthracene (B[a]A), chrysene (CHRY), benzo[b]fluoranthene (B[b]F), benzo[k]fluoranthene (B[k]F), benzo[a]pyrene (B[a]P), benzo[g,h,i]perylene (B[ghi]P), indeno[1,2,3-c,d]pyrene (IND), and dibenz[a,h]anthracene (D[ah]A).

A comparison of the content of polycyclic aromatic hydrocarbons in the tested soil (Soil AB) inoculated with (a) biopreparation A1; (b) biopreparation A1 with the addition of γ-PGA after 2 (Soil A2/Soil B2), 4 (Soil A4/Soil B4) and 6 (Soil A6/Soil B6) months of the biodegradation process is presented in Figure 5.

The chromatographic analyses performed showed that the addition of γ-PGA to a biopreparation has a positive effect on the efficiency of the biodegradation process of petroleum hydrocarbons. As a result of inoculation of soil AB with the biopreparation A1 with the addition of γ-PGA over 6 months, higher degrees of biodegradation of all analyzed analytes were obtained than as a result of inoculation with the biopreparation A1. It can therefore be concluded that γ-PGA supports the decomposition of both aliphatic and aromatic hydrocarbons in the presence of the biopreparation. High values of biodegradation degrees of particular groups of petroleum hydrocarbons (aliphatic hydrocarbons, BTEX, WWAs) confirm the supposition that the biopreparation consisted of *Dietzia* sp. *IN133*, *Gordonia* sp. *IN138 Mycolicibacterium frederiksbergense IN53*, *Rhodococcus erythropolis IN119*, *Rhodococcus* sp. *IN136* and *Pseudomonas* sp. *IN132*. in combination with gamma polyglutamic acid, it can be used as an inoculum in bioaugmentation procedures. The use of this type of inoculation allows for the effective biodegradation of petroleum pollutants present in contaminated soils, and is also safe for the environment (Section 3.3). The summary of the content of particular groups of petroleum hydrocarbons in the initial soil AB and after the completion of the biodegradation process (6 months) is provided in Table 3.

The course of the biodegradation process of petroleum pollutants (TPH, BTEX, PAHs) during the AB soil inoculation process with biopreparation A1 and biopreparation A1 with the addition of γ-PGA is described by Equation (2). The individual coefficients of Equation (2) are summarized in Table 4.

The first order biodegradation constants (k) during inoculation of soil AB with biopreparation A1 for aliphatic hydrocarbons are at the following level: TPH (0.0058 d^−^^1^), Σ n-C_9_–n-C_22_ (0.0113 d^−^^1^), Σ n-C_23_–n-C_40_ (0.0041 d^−^^1^), and for mono and polycyclic aromatic hydrocarbons they are, respectively: BTEX (0.0072 d^−^^1^) and PAHs (0.0037 d^−^^1^). The presented results prove the biodegradability of both aliphatic and aromatic hydrocarbons. The biodegradation rate of polycyclic aromatic hydrocarbons is directly proportional to the number of rings in the molecule and the size of their concentration in the soil (Table 4) The first order biodegradation constants (k) during the inoculation of soil AB with biopreparation A1 with the addition of γ-PGA visibly increase at the level of: TPH (0.0083 d^−^^1^), Σ n-C_9_–n-C_22_ (0.0186 d^−^^1^), Σ n-C_23_–n-C_40_ (0.0069 d^−^^1^), BTEX (0.0112 d^−^^1^) and PAHs (0.0046 d^−^^1^) (Table 4). The obtained results prove that the addition of γ-PGA increases the degree of biodegradation of both aliphatic and aromatic hydrocarbons.

### 3.3. Toxicological Assessment

Pollution of the natural environment with petroleum substances such as TPH, BTEX or PAHs contributes to the constant deterioration of the health of living organisms. Therefore, a comprehensive assessment of soil quality should be based not only on the concentration of selected physico-chemical parameters, but also include a bioindication analysis assessing its toxicity to living organisms. The assessment of the effectiveness of the bioremediation treatments used was supplemented with toxicological monitoring aimed at determining the impact of petroleum pollutants (TPH, BTEX, PAHs) and intermediate metabolites formed during remediation treatments on soil biocenosis based on the performed toxicological tests. Toxicological tests allow for the simultaneous determination of the harmful effect of all substances in the tested sample on selected living organisms, taking into account the interactions between all elements of the tested system. The use of living organisms as bioindicators belonging to various taxonomic groups (bacteria, crustaceans and higher plants) representing all trophic groups (producers, consumers and decomposers) allowed for a comprehensive assessment of the effectiveness of the treatment of soil contaminated with petroleum substances. The results of Phytotoxkit^TM^, Ostracodtoxikit F^TM^ and Microtox SPT toxicology tests is listed in Table 5.

On the basis of the results of toxicological tests, it is clearly visible that the initial soil AB was toxic to the tested species of plants, animals and microorganisms.

In the initial AB soil, the observed seed germination inhibition ranged from 20% to 40% depending on the tested organism, while for each of the analyzed plants, more than 74% of root growth was inhibited. *Lepidium sativum* turned out to be the most sensitive to pollutants from the group (TPH, BTEX, PAHs), while *Sorghum saccharatum* was characterized by the highest resistance. Both bioaugmentation variants used improved the quality of the soil by reducing its phytotoxicity compared to the initial AB soil, but differed in the effectiveness of phytotoxicity reduction. The soil inoculated with the biopreparation A1 with the addition of γ-PGA was characterized by lower phytotoxicity after 6 months of the purification process, for which the inhibition of root growth decreased by 69.58–74.35%, while the inhibition of seed germination decreased by 20–40% compared to the starting soil AB. Slightly lower values of the decrease in root growth inhibition after 6 months of biodegradation were observed in the soil inoculated with the biopreparation A1 and amounted to 61.25–64.93% in relation to the AB soil. In general, it can be concluded that the greater the reduction in the content of petroleum hydrocarbons during the biodegradation process, the less phytotoxic the properties of the soil are.

The Ostracodtoxikit F^TM^ direct contact test carried out on the soil before the inoculation treatment showed the toxic properties of the tested soil, as evidenced by the high mortality of *Heterocypris incongruens* (83.33%) and inhibition of the growth of ostracods by 70.60%. The successive reduction of the content of petroleum pollutants through the conducted inoculation procedures resulted in the reduction of the toxicity of the tested soil for spouses, which improved the survival and growth of *Heterocypris incongruens*. The tests performed after 180 days of biodegradation by inoculation with the biopreparation A1 with the addition of γ-PGA showed a 90.00% survival effect of *Heterocypris incongruens*, while the average length of the tested organisms after the end of the test was 764 μm. The second examined parameter, growth inhibition of ostracods, was 4.99%. Compared to the inoculation with the biopreparation A1, the use of γ-PGA supplemented the survival effect of ostracods by 8.33% and the decrease in the average growth inhibition of *Heterocypris incongruens* by 5.12%.

The soil toxicity tests at the trophic level of the decomposers were carried out using the Microtox^®^ SPT test (for the solid phase), which allowed for direct contact of luminescent bacteria (*Vibrio fischeri*) with the tested soil sample. Thanks to this, it is possible to detect not only water-soluble substances, but also hydrophobic and lipid compounds. Acute toxicity expressed in toxic units (TU) was high in the initial AB soil (Table 4). Conducting the inoculation treatment with the biopreparation A1 and the biopreparation A1 with the addition of γ-PGA resulted in a reduction of the toxicity of the tested soil for microorganisms. Compared to the initial AB soil, inoculation with the biopreparation decreased soil toxicity by 22.8%, and the inoculation with the biopreparation A1 supplemented with γ-PGA by 28%.

**Figure 6:**
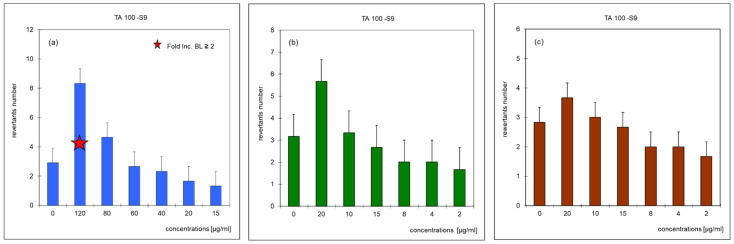
Influence of TPH, BTEX and PAHs concentration of the revertants number (*n* = 3, *p* < 0.05): (**a**) crude soil (Soil AB); (**b**) Soil A6—soil after a 6-month period of biopreparation A1 inoculation; (**c**) Soil B6—soil after a 6-month period of biopreparation A1 with the addition of γ-PGA inoculation.

In order to determine the presence of mutagenic and carcinogenic compounds in soil samples: raw soil (Soil AB), and after a 6-month inoculation period with biopreparation A1 (Soil A) and biopreparation A1 with addition of γ-PGA (Soil B) Ames test (Figure S2) was run. The study used the TA-100 strain, which is a standard strain of *Salmonella typhimirium*. Its mutations occur on the histidine operon, which is unable to synthesize amino acids. It was noticed that the use of the microsomal S9 fraction as a metabolic activator of promutagenes had a slight effect on the increase in the number of reversible mutations. The results of the tested soil samples are shown in Figure 6 as changes in the number of revertants indicated depending on the concentration of pollutants. In the case of a sample of raw soil (Soil AB) found to be mutagenic, the mutagenicity coefficient was 6.15. After biodegradation of petroleum pollutants as a result of inoculation with the biopreparation A1 (6 months) (soil A6), the mutagenicity coefficient decreased gradually to 2.15, and after inoculation with the biopreparation with the addition of γ-PGA (Soil B6) to 1.54 (Figure 6). The Soil A6 and Soil B6 samples showed no mutagenic properties as the number of non-histidine induced revertants was only slightly higher than the number of spontaneous mutations present in the control sample.

The conducted toxicological tests confirmed the correlation between the effectiveness of removing petroleum pollutants from the soil matrix and the reduction of its toxicity. In the soil inoculated with the biopreparation with the addition of γ-PGA, a greater reduction in the content of TPH, BTEX and PAHs was observed, and thus a greater improvement in the quality of the tested soil in terms of its toxicity, than in the soil inoculated with the biopreparation A1.

The susceptibility of petroleum substances to microbial degradation depends mainly on their chemical structure. Aliphatic hydrocarbons are usually more easily degraded than that aromatic, long-chain paraffins easier than short-chain, saturated compounds easier than unsaturated and unbranched faster than branched. Cycloalkanes, carbon polycyclic hydrocarbons, compounds with naphthenic-aromatic structures, asphaltenes and other heavy fractions are usually less biodegradable [4]. Biodegradation with the use of biopreparations developed on the basis of indigenous microorganisms capable of decomposing a wide spectrum of pollutants is currently considered one of the most efficient and environmentally friendly techniques for land remediation. The ability of microorganisms to catabolize various types of organic pollutants is a necessary condition for the proper conduct of the biodegradation process, which is why it has been extensively studied and discussed by researchers from around the world. The challenge now is to improve the efficiency of land remediation processes by biological methods. An interesting solution seems to be the use of various types of additives enriching biopreparations. It is important that the additives used are not toxic. Such an additive in the case of remediation of soils contaminated with petroleum hydrocarbons can be γ-PGA. The conducted research on the use of the γ-PGA polyamide polymer as an additive to a biopreparation A1 has shown that it increases the efficiency of the biodegradation process of petroleum pollutants in the soil matrix. This is due to the special structure of γ-PGA, which makes this compound less susceptible to the action of proteolytic enzymes, which protects the bacterial cell against microbial attack. Moreover, γ-PGA is characterized by strong hygroscopic properties, which in turn allows to maintain favourable conditions for the treatment process of contaminated soils, even in unfavourable weather conditions (drought). It is estimated that in the form of a hydrogel, γ-polyglutamic acid is able to bind water molecules in an amount up to 3500 times its mass [116]. Moreover, depending on the pH of the environment in which it is located, it can be highly soluble in water (pH ≥ 6.5) or in organic solvents (pH = 2). Due to these properties, as well as good biodegradability, lack of toxicity and immunogenicity, research into the use of γ-polyglutamic acid in various fields of science is constantly being conducted [117]. In environmental engineering, γ-PGA has been used as a compound that binds harmful substances, which is used in the remediation of soils contaminated with heavy metals. Literature reports indicate that γ-PGA may be released into the environment to sequester toxic metal ions, reduce the salt concentration in the soil environment, provide a carbon source as food for microorganisms, and protect against adverse conditions. In addition, γ-PGA can improve biofilm formation and aid the absorption of essential nutrients from the environment [118]. Research on the biodegradation of petroleum hydrocarbons carried out by the *ex-situ* prism method has shown that γ-polyglutamic acid can be used as an additive to biopreparations in order to increase the efficiency of treating contaminated soils. In addition, research studies have shown that γ-PGA improves the bioavailability of nitrogen in the soil due to microbial transmission in the soil, which increases the possibility of obtaining data by hydro massage. Research by Zhang and his team [119] The study result that γ-PGA may stem from an increase in root biomass. In addition, γ-PGA can be used by plants as a source of nitrogen or carbon, we support their construction. The chromatographic analyses carried out showed that the addition of γ-PGA to the biopreparation A1 increased the biodegradation efficiency of TPH by 13.18%, BTEX by 10.11%, and PAHs by 12.32%. In addition, the toxicological tests carried out showed that the addition of γ-PGA is not toxic and does not increase soil toxicity during the treatment process, and even has a positive effect on its quality. This statement is confirmed by the results of the Phytotoxkit^TM^, Ostracodtoxikit F^TM^, Microtox SPT, and Ames tests carried out during biodegradation, which indicate a greater reduction in the toxicity level of soil inoculated with the biopreparation A1 with the addition of γ-PGA. Moreover, the research carried out with the use of the Phytotoxkit^TM^ test confirms the research of Zhang and his team [119] on the effect of the addition of γ-PGA on the biomass of plant roots. Greater root length of *Sorghum saccharatum*, *Lepidium Sativum* and *Sinapis alba* plants was observed in soil inoculated with biopreparation A1 with the addition of γ-PGA than in soil inoculated with biopreparation A1.

## 4. Conclusions

The aim of the study was to evaluate the possibility of using γ-PGA as an additive to the biopreparation A1 in the process of biodegradation of petroleum hydrocarbons. During the 6-month research, the effectiveness of the biodegradation process of petroleum hydrocarbons (TPH, BTEX, PAHs) was determined as a result of inoculation of contaminated soil with biopreparation A1 and biopreparation A1 with the addition of γ-PGA. Biodegradation studies carried out by the *ex-situ* prism method with the use of 2 inoculation methods have shown that the addition of γ-PGA increases the efficiency of decomposition of TPH, BTEX and PAHs. The higher microbiological activity observed during respirometric tests and the conducted chromatographic analyses showed that the addition of γ-PGA to the biopreparation increased the biodegradation efficiency of TPH by 13.18%, BTEX by 10.11%, and PAHs by 12.32% compared to the biopreparation A1. The introduction of a set of selected toxicological tests allowed for the observation of changes in the impact of the concentration of petroleum pollutants on various types of bioindicators (plants, animals, microorganisms) representing all trophic groups during the biodegradation process of TPH, BTEX and PAHs. The conducted toxicological tests confirmed the correlation between the effectiveness of removing petroleum pollutants from the soil matrix and the reduction of its toxic effect on the tested bioindicators. This is evidenced by the values of toxicity indicators in the Phytotoxkit^TM^ (germination inhibition, root growth inhibition), Ostracodtoxkit F^TM^ (mortality, growth inhibition) and Microtox SPT (TU) tests, which after the completion of the biodegradation process of petroleum hydrocarbons were lower in the soil inoculated with A1 biopreparation with the addition of γ-PGA rather than A1 biopreparation only. Research on the acceleration of soil bioremediation processes using microorganisms is now an important aspect of environmental engineering. The use of gamma polyglutamic acid as an additive to a biopreparation based on autochthonous bacteria can significantly improve the efficiency of biodegradation processes. Satisfactory results of research carried out in *ex-situ* conditions create the prospect of adopting the method of cleaning soils contaminated with petroleum hydrocarbons with the use of biopreparation A1 with the addition of γ-PGA on an industrial scale.

## Figures and Tables

**Table 1:** Genes involve in petroleum hydrocarbon degradation, found in genomes for the closest relatives of all strains.

Stain	The Closest Relative Based on 16S rRNA Accession Number, (% of Identity) *	The Closest Relative for Which the Genome Sequence Is Available in NCBI GenBank, Accession Number, (% of Identity) *	Putative Gene Encoding for the Enzymes Degrading Hydrocarbons
*Dietzia* sp. *IN133*	*Dietzia* sp. JS16-p6b CP024869 99.93%	*Dietzia* sp. JS16-p6b CP024869 99.93%	Alkane 1-monooxygenase (4 copies), biphenyl 2,3-dioxygenase, (2 copies), 2,3-dihydroxybiphenyl 1,2-dioxygenase (2 copies)
*Gordonia* sp. *IN138*	*Gordonia terrae* RL-JC02 CP049836 99.65%	*Gordonia terrae* RL-JC02 CP049836 99.65%	Alkane 1-monooxygenase, pentachlorophenol monooxygenase, naphthalene 1,2-dioxygenase subunit alpha (2 copies), 2,3-dihydroxybiphenyl 1,2-dioxygenase (2 copies)
*Mycolicibacterium frederiksbergense* *IN53*	*Mycolicibacterium frederiksbergense* DSM 44346 (typical strain) NR_025393.1 99.58%	*Mycolicibacterium frederiksbergense* LB 501T 99.8%	Alkane 1-monooxygenase (2 copies), pentachlorophenol monooxygenase, naphthalene 1,2-dioxygenase subunit alpha (2 copies), 2,3-dihydroxybiphenyl 1,2-dioxygenase (2 copies)
*Rhodococcus erythropolis* *IN119*	*Rhodococcus erythropolis* KD-1 CP050124 99.42%	*Rhodococcus erythropolis* KD-1 CP050124 99.42%	Alkane 1-monooxygenase (5 copies), pentachlorophenol monooxygenase, cyclohexanone monooxygenase (2 copies), biphenyl 2,3-dioxygenase (2 copies), 2,3-dihydroxybiphenyl 1,2-dioxygenase (2 copies)
*Rhodococcus* sp. *IN136*	*Rhodococcus fascians* KHO60 MN099376 99.71%	*Rhodococcus fascians* D188 CP015235 99.71%	Alkane 1-monooxygenase (3 copies), methane monooxygenase 2,4-dichlorophenol 6-monooxygenase, 1 element of 1,2-benzene dioxygenase system, 2,3-dihydroxybiphenyl 1,2-dioxygenase
*Pseudomonas* sp. *IN132*	*Pseudomonas citronellolis* PY-1 MH685460 99.49%	*Pseudomonas citronellolis* SJTE-3 CP015878 99.49%	Alkane 1-monooxygenase, phenol 2-monooxygenase, 2,4-dichlorophenol 6-monooxygenase, naphthalene 1,2-dioxygenase, biphenyl 2,3-dioxygenase, 2,3-dihydroxybiphenyl 1,2-dioxygenase

* the presented data concerns the data gathered in the NCBI GenBank database (November 2021).

**Table 2:** Hydrocarbon-degrading capabilities of bacterial strains comprising the biopreparation A1.

Stain	NCBI Accession Number	nC_18_H_38_	iso C_19_H_40_	TOL + XYL	NAP	ANTH	PHEN	FLU	FLTH	PYR
*Dietzia* sp. *IN133*	KT923300	+	+	+	+	−	−	−	−	−
*Gordonia* sp. *IN138*	KT923297	+	+	+	+	+/−	−	−	−	−
*Mycolicibacterium frederiksbergense* *IN53*	JN572675	+	+	−	+	+	+	−	−	−
*Rhodococcus erythropolis* *IN119*	KT923331	+	+	+	+	−	−	−	−	−
*Rhodococcus* sp. *IN136*	KT923330	+	+	−	+	−	−	−	+	−
*Pseudomonas* sp. *IN132*	KT923299	+	+	+	+	+/−	+	+/−	+/−	+/−

nC_18_H_38_: n-octane iso-C_19_H_40_: pristane, TOL: toluene, XYL: mixture of xylenes NAP: naphtalene, ANTH: anthracene, PHEN: phenanthrene FLU: fluorene, FLTH: fluoranthene, PYR: pyrene, +: growth, −: no growth +/−: ambiguous observation.

**Table 3:** Summary of the content of petroleum hydrocarbons (TPH, BTEX, PAHs) in soil AB and after the completion of the biodegradation process with the use of biopreparation A1 (Soil A6) and biopreparation A1 with the addition of γ-PGA (Soil B6), (repetition number *n* = 7–10, *p* < 0.05).

Hydrocarbons	Content ± SD (mg/kg d.w. Soil)		
	Initial Soil AB	After 180 Days	
		Soil A6 (Inoculated with Biopreparation)	Soil B6 (Inoculated with Biopreparation with PGA)
TPH	19,774.23 ± 988.71	6717.33 ± 671.73	4111.47 ± 411.15
Unidentified aliphatic hydrocarbons	10,196.10 ± 509.81	4130.44 ± 413.04	2871.44 ± 287.14
∑ n-C_9_–n-C_21_	5356.69 ± 535.67	600.42 ± 120.08	154.50 ± 30.90
∑ n-C_22_–n-C_30_	3027.63 ± 302.76	1149.09 ± 229.82	451.96 ± 90.39
∑ n-C31–n-C_40_	683.31 ± 68.33	447.55 ± 89.51	276.31 ± 55.26
BTEX	17.45 ± 1.75	3.48 ± 0.70	1.71 ± 0.34
benzene	3.56 ± 0.53	0.29 ± 0.09	0.15 ± 0.05
toluene	1.11 ± 0.17	0.04 ± 0.02	0.01 ± 0.004
ethylobenzene	0.47 ± 0.09	0.03 ± 0.01	0.01 ± 0.004
xylenes	12.31 ± 1.23	3.11 ± 0.62	1.54 ± 0.31
PAHs	27.03 ± 2.70	16.52 ± 1.62	13.19 ± 1.32
∑ two-ring PAHs	1.37 ± 0.27	0.39 ± 0.12	0.26 ± 0.08
∑ three-ring PAHs	2.65 ± 0.40	0.96 ± 0.14	0.76 ± 0.11
∑ four-ring PAHs	15.19 ± 1.52	9.41 ± 0.94	7.47 ± 0.75
∑ five-ring PAHs	6.01 ± 0.60	4.41 ± 0.44	3.55 ± 0.36
∑ Six-ring PAHs	1.81 ± 0.36	1.36 ± 0.27	1.15 ± 0.23

**Table 4:** Coefficients of first-order mathematical model describing TPH, BTEX and PAHs hydrocarbons group biodegradation in soil AB. Measurement repetition number *n* = 7–10, *p* < 0.05.

Petroleum Pollutants Group	k [d^−1^]		(C/C_H_)_0_		Determination Coefficient (r^2^)	
	Inoculated Biopreparation A1	Inoculated Biopreparation A1 with γ-PGA	Inoculated Biopreparation A1	Inoculated Biopreparation A1 with γ-PGA	Inoculated Biopreparation A1	Inoculated Biopreparation A1 with γ-PGA
TPH	0.0058 ± 0.0003	0.0083 ± 0.0005	3763 ± 298	3639 ± 201	0.9944	0.9913
∑ n-C_9_–n-C_22_	0.0113 ± 0.0007	0.0186 ± 0.0011	1177 ± 95	10278 ± 89	0.9912	0.9939
∑ n-C_23_–n-C_40_	0.0041 ± 0.0002	0.0069 ± 0.0006	634 ± 23	603 ± 16	0.9931	0.9916
BTEX	0.0072 ± 0.0004	0.0112 ± 0.007	3.403 ± 0.182	3.198 ± 0.16	0.9956	0.9906
PAHs	0.0037 ± 0.002	0.0046 ± 0.002	5.095 ± 0.291	4.844 ± 0.27	0.9897	0.9874
∑ two-ring PAHs	0.0061 ± 0.0004	0.0077 ± 0.0004	0.192 ± 0.061	0.176 ± 0.056	0.9953	0.9894
∑ three-ring PAHs	0.0053 ± 0.0003	0.0062 ± 0.0004	0.529 ± 0.032	0.314 ± 0.024	0.9877	0.9842
∑ four-ring PAHs	0.0031 ± 0.0002	0.0036 ± 0.0002	2.964 ± 0.158	2.366 ± 0.128	0.9847	0.9789
∑ five-ring PAHs	0.0018 ± 0.0002	0.0021 ± 0.0002	1.870 ± 0.091	1.560 ± 0.067	0.9961	0.9909
∑ Six-ring PAHs	0.0009 ± 0.0001	0.0011 ± 0.0002	0.352 ± 0.019	0.322 ± 0.015	0.9842	0.9882

**Table 5:** The results of Phytotoxkit^TM^, Ostracodtoxikit F^TM^ and Microtox SPT toxicology tests.

Tested Organism	Parameter Measured	Control Soil	Soil AB	Soil A3	Soil B3	Soil A6	Soil B6
Test Phytotoxkit^TM^							
*Sorghum saccharatum*	germination [%]	100	80	100	100	100	100
	average root length [mm]	48	11.4	27.6	31.7	40.8	44.8
	root growth inhibition mean [%]	0	76.25	42.50	33.96	15.00	6.67
*Lepidium Sativum*	germination [%]	100	60	80	90	100	100
	average root length [mm]	69	11.6	35.1	44.6	56.4	62.9
	root growth inhibition mean [%]	0	83.19	49.13	35.36	18.26	8.84
*Sinapis alba*	germination [%]	100	70	90	100	100	100
	average root length [mm]	80	20.2	47.5	55.8	68.4	75.5
	root growth inhibition mean [%]	0	74.75	40.63	30.25	14.5	5.63
**Test Ostracodtoxikit F^TM^**							
*Heterocypris incongruens*	average mortality [%]	4	81.67	43.33	36.67	18.33	10.00
	average growth inhibition [%]	-	70.6	34.98	29.01	10.11	4.99
**Test Microtox SPT**							
*Vibrio fischeri*	*TU*	0	32.3	16.5	14.2	9.4	4.3

## Data Availability

Not applicable.

## Supplementary Materials

The following supporting information can be downloaded at: https://www.mdpi.com/article/10.3390/ma15020400/s1. Figure S1: Content (%) of individual components of petroleum pollutants in the soil, Ecotoxicological analyses –description of the methodology. Figure S2: Scheme of Ames Test conduct.
