# Supplementary material for: Evaluation of the Effectiveness of the Biopreparation in Combination with the Polymer γ-PGA for the Biodegradation of Petroleum Contaminants in Soil
Source: Materials (Basel). 2022 Jan 6;15(2):400. doi: 10.3390/ma15020400 (PMC8778143; doi:10.3390/ma15020400)
Supplement: Supplementary file 1 [file materials-15-00400-s001.zip › materials-1537141-supplementary.pdf]

materials

MDPI

Suplementary materials

Evaluation of the Effectiveness of the Biopreparation in Combination with the Polymer PGA for the Biodegradation of Soils Contaminated with Petroleum Hydrocarbons

Katarzyna Wojtowicz [1,*], Teresa Steliga [1], Piotr Kapusta [1], Joanna Brzeszcz [1] and Tomasz Skalski [2]

[1] Oil and Gas Institute—National Research Institute, Lubicz 25 A, 31-503 Krakow, Poland; teresa.steliga@inig.pl (T.S.); piotr.kapusta@inig.pl (P.K.); joanna.brzeszcz@inig.pl (J.B.)
[2] Biotechnology Centre, Silesian University of Technology, Krzywoustego 8, 44-100 Gliwice, Poland; tomasz.skalski@polsl.pl
* Correspondence: katarzyna.wojtowicz@inig.pl

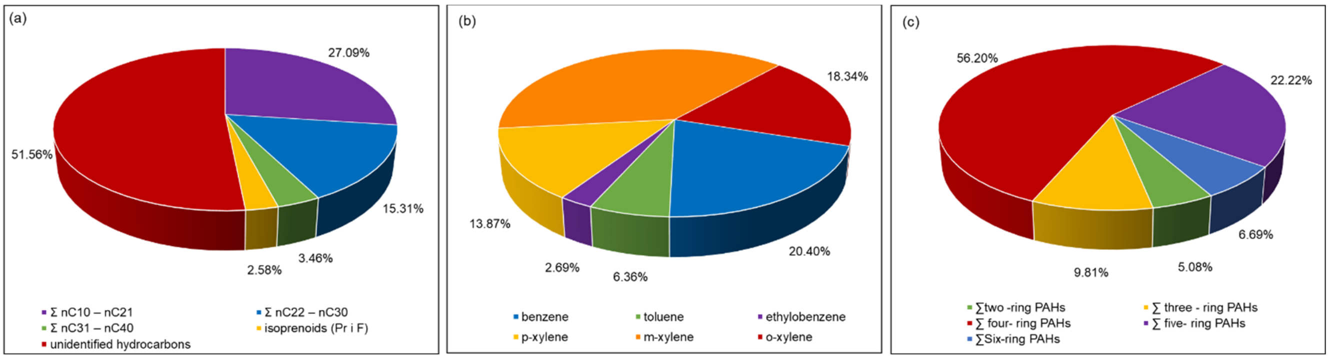

Figure S1. Content (%) of individual components of petroleum pollutants in the soil. (**a**) TPH; (**b**) BTEX; (**c**) PAHs.

Ecotoxicological analyses–description of the methodology

Phytotoxkit™ test (MicroBioTests Inc., Nazareth, Belgium)

Chronic toxicity assessment test *Phytotoxkit™* is based on the evaluation of germination and early growth of plants (root elongation inhibition measurement. Three types of plants selected according to germination rate and root growth rate are used in the test, which allows making a complete determination in 3 days of incubation: monocotyledonous - sorghum *(Sorghum saccharatum)*, and dicotyledonous-cress *(Lepidium sativum)* and white mustard *(Sinapis alba)*. The determination process was carried out using three repetitions for each test plant. The tests were performed on transparent polystyrene test plates. *Incubation conditions:* temperature T = 25 °C in darkness, incubation time t = 72 h. *Test reaction:* inhibition of germination and early root growth [1–3].

The inhibition in seed germination and root growth was calculated according to a following equation:

$$I = \frac{(A - B)}{B} \cdot 100\%$$

where: I—inhibition (%), A—seed germination or root length in control soil, B—seed germination or root length in test soil

Ostracodtoxkit(F)™ (MicroBioTest Inc., Nazareth, Belgium)

Ostracodtoxkit(F) test [4] belongs to direct contact tests of chronic toxicity estimation with the use of crustaceans *Heterocypris incongruens*. The direct contact test is done with the application of young bottom crustaceans (*Heterocypris incongruens*), which hatched from cysts during 52 h (according to the producer's procedure). The test is carried out on 6-hole polystyrene microplates. A standardised nutrient is an algae suspension, which is applied to the suspension in portions of 2 ml. Portions of 10 ostracods are added to each cell of the microplate. 0.5 g of a control soil is applied to the row A, whereas 0.5 g of the soil sample is added to the consecutive rows. Incubation lasts for 6 days in a temperature of 25 °C. Results reading consists of estimation of life microforms amount in each hole of the microplate and measurement of their length. Growth inhibition of *H. incongruens* was calculated as

$$GI = 100 - \left(\frac{A}{B} \cdot 100\%\right)$$

where: A—ostracods length in tested soil, B—ostracods length in reference soil.

Microtox® Solid Phase Test (SDI, Newark, DE, USA)

Microtox Test created in the USA in 1979 as the first bioindication test, combines typical bioindication and analytic precision. Luminescence bacteria *Vibro fisch*eri, which uses about 10% of its metabolism in order to emit light, was used as a bioindicator. In the presence of toxical substances, there is decrease in luminescence and increase in general toxicity of a sample. In an electron transportation system of the bacteria, lucipherasa enzyme (alcane oxygenaze) catalyses oxidation of a reduced substrate (reduced flavine mononucleotide, riboflavin phosphate or flavine adenin dinucleotide) and during this process luminescence, which can be measured with a photometer, takes place. The obtained substrates of this reaction are oxygen and long-chain aldehyde. In the presence of substances that have negative influence on cell metabolism, the decrease in luminescence of bacteria is immediate.

Test Microtox®, produced by SDI Company (USA), enables a direct contact of luminescence bacteria *Vibro fischeri* with a wastewater sample, which leads not only to determination of substances dissolved in water, but also to recognition of lipophilicity systems and poorly dissolved systems in water. Lyophilised bacteria *Vibro fischeri* can be stored in a period of a year in temperature of -20°C and then used for testing immediately after suspending in deionised water. A tests with serial dilutions of the wastewater were done according to a standard procedure and results were obtain in Delta Tox analyser. In this analysis, the concentration of wastewater, which reduces the luminescence by 50%, was determined (half maximal effective concentration, EC_{50}). In this presentation a lower of EC_{50} denotes a higher toxicity. To simplify this interpretation, $EC_{50'}$ values were converted to Toxicity Units (TU) in which a higher value denotes a higher toxicity according to the following formula [5–9].

$$\mathrm{TU} = \frac{1}{EC_{50}} \cdot 100$$

AMES (Muta-ChromoPlate™ Kit (EBPI, Mississauga, ON, Canada).

Numerous petroleum contaminants and metabolites created in biodegradation of pollutants include carcinogenic compounds. In order to estimate potentially mutagenic properties of the wastewater, Ames Test was applied. It enables determination of reverse mutations from histidine auxotrophy to prototrophy in mutants, which are test strains of *Salmonella typhimurium*. The test strains of the bacteria can be characterised by various types of mutation in genes, which are responsible for histidine synthesis. Therefore, the presence of histidine in a substratum is required in order to provide the growth of the strains. Generally, TA98 and TA100 strains are used [9–11]. They include proper mutations consisting of so-called reading frame displacement, pair base substitution and two mutagenes (2-nitro-fluoren and sodium

azide). The test strains are mutants, which are unable to histidine synthesis. A mutagenous substance causes reversion in mutation and histidine synthesis can take place, which results in appearance of revertants colonies in the histidine-free basis.
In order to lead Ames Test, a high-tech microplate AMES MPF test was applied. It can be used for detection of genotoxic activity of water contaminated with petroleum substances and metabolites, which are the effects of biodegradation. Delivered microforms of T-98 and T-100 *Salmonella typhimurium* undergo strict quality control (genotype and phenotype). The test results in colour reaction, where yellow means reverse mutation, whereas scarlet—the lack of mutation.

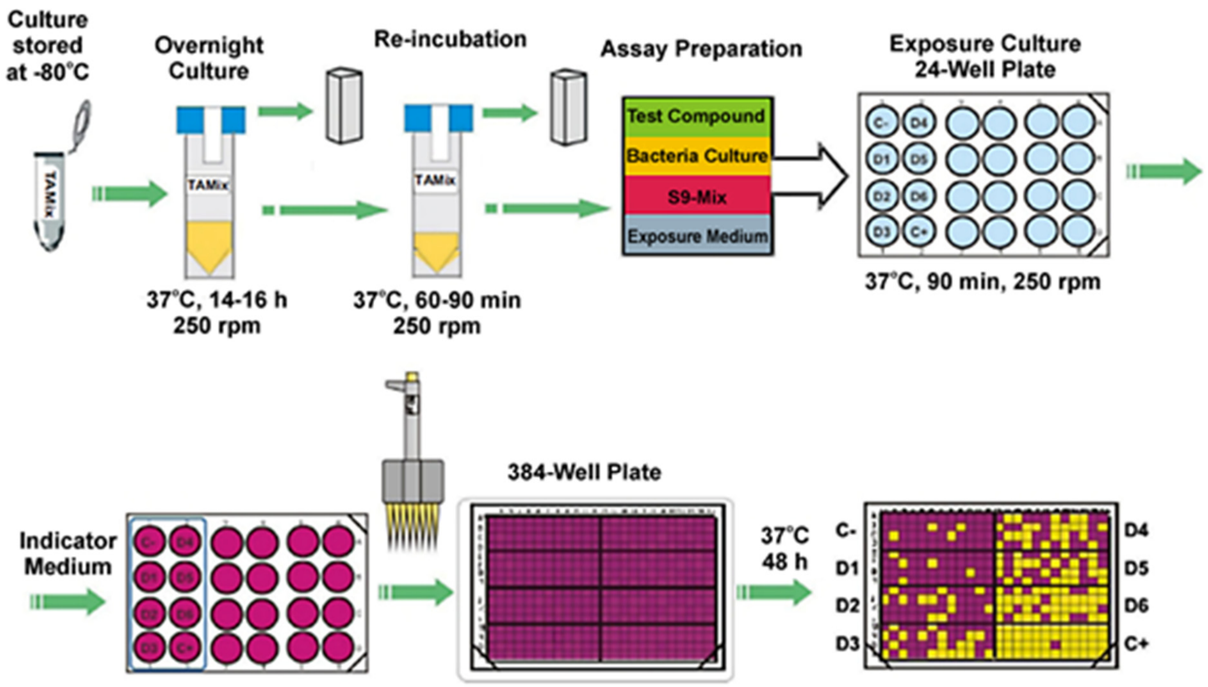

Figure S2. Scheme of Ames Test conduct [12].

Petroleum contaminants from the samples were extracted with the use of dichloromethane, which was then vaporised and the obtained deposit was dissolved in dimethylosulfoxide (DMSO) [10]. As an activator, S9 microsomal fraction from a rat liver was used. The role of the activator is to transform the tested substance, which leads to disclosure of its potentially mutagenous properties.

References

1. Baran, A.; Tarnawski, M. Phytotoxkit/Phytotestkit and Microtox® as Tools for Toxicity Assessment of Sediments. *Ecotoxicology and Environmental Safety* **2013**, *98*, 19–27, doi:10.1016/j.ecoenv.2013.10.010.
2. Blinova, I.; Bityukova, L.; Kasemets, K.; Ivask, A.; Käkinen, A.; Kurvet, I.; Bondarenko, O.; Kanarbik, L.; Sihtmäe, M.; Aruoja, V.; et al. Environmental Hazard of Oil Shale Combustion Fly Ash. *Journal of Hazardous Materials* **2012**, *229–230*, 192–200, doi:10.1016/j.jhazmat.2012.05.095.
3. Mamindy-Pajany, Y.; Hamer, B.; Roméo, M.; Géret, F.; Galgani, F.; Durmiši, E.; Hurel, C.; Marmier, N. The Toxicity of Composted Sediments from Mediterranean Ports Evaluated by Several Bioassays. *Chemosphere* **2011**, *82*, 362–369, doi:10.1016/j.chemosphere.2010.10.005.
4. Niyommaneerat, W.; Nakajima, F.; Tobino, T.; Yamamoto, K. Development of a Chronic Sediment Toxicity Test Using the Benthic Ostracod Heterocypris Incongruens and Their Application to Toxicity Assessments of Urban Road Dust. *Ecotoxicology and Environmental Safety* **2017**, *143*, 266–274, doi:10.1016/j.ecoenv.2017.05.011.
5. Lima, T.M.S.; Procópio, L.C.; Brandão, F.D.; Leão, B.A.; Tótola, M.R.; Borges, A.C. Evaluation of Bacterial Surfactant Toxicity towards Petroleum Degrading Microorganisms. *Bioresource Technology* **2011**, *102*, 2957–2964, doi:10.1016/j.biortech.2010.09.109.

6. Adams, R.H., Kanga-Leyva, K., Guzmán-Osorio, F.J., Escalante-Espinoza, E, .2011. Comparison of moisture management methods for the bioremediation of hydrocarbon contaminated soil. *African Journal of Biotechnology* **2011**, *10*, 394-404.
7. Foucault, Y.; Durand, M.-J.; Tack, K.; Schreck, E.; Geret, F.; Leveque, T.; Pradere, P.; Goix, S.; Dumat, C. Use of Ecotoxicity Test and Ecoscores to Improve the Management of Polluted Soils: Case of a Secondary Lead Smelter Plant. *Journal of Hazardous Materials* **2013**, *246–247*, 291–299, doi:10.1016/j.jhazmat.2012.12.042.
8. Oleszczuk, P.; Jośko, I.; Kuśmierz, M.; Futa, B.; Wielgosz, E.; Ligęza, S.; Pranagal, J. Microbiological, Biochemical and Ecotoxicological Evaluation of Soils in the Area of Biochar Production in Relation to Polycyclic Aromatic Hydrocarbon Content. *Geoderma* **2014**, *213*, 502–511, doi:10.1016/j.geoderma.2013.08.027.
9. Kamber, M.; Fluckiger-Isler, S.; Engelhardt, G.; Jaeckh, R.; Zeiger, E. Comparison of the Ames II and Traditional Ames Test Responses with Respect to Mutagenicity, Strain Specificities, Need for Metabolism and Correlation with Rodent Carcinogenicity. *Mutagenesis* **2009**, *24*, 359–366, doi:10.1093/mutage/gep017.
10. Steliga, T.; Jakubowicz, P.; Kapusta, P. Changes in Toxicity during in Situ Bioremediation of Weathered Drill Wastes Contaminated with Petroleum Hydrocarbons. Bioresource Technology 2012, 125, 1–10, doi:10.1016/j.biortech.2012.08.092.
11. Vijay, U.; Gupta, S.; Mathur, P.; Suravajhala, P.; Bhatnagar, P. Correction: Microbial Mutagenicity Assay: Ames Test. *BIO-PROTOCOL* **2018**, *8*, doi:10.21769/BioProtoc.3016.
12. Available online: http://www.tigret.eu/images/stories/produkty/Toksykologia/Ames_MPF_PL_20191206.pdf (accessed on 15 March 2021).
